# When *Wine* and *Apple* Both Help the Production of *Grapes*: ERP Evidence for Post-lexical Semantic Facilitation in Picture Naming

**DOI:** 10.3389/fnhum.2018.00136

**Published:** 2018-04-10

**Authors:** Grégoire Python, Raphaël Fargier, Marina Laganaro

**Affiliations:** Faculty of Psychology and Educational Science, University of Geneva, Geneva, Switzerland

**Keywords:** semantic facilitation, semantic priming, ERP, language production, picture naming, response selection

## Abstract

**Background**: Producing a word in referential naming requires to select the right word in our mental lexicon among co-activated semantically related words. The mechanisms underlying semantic context effects during speech planning are still controversial, particularly for semantic facilitation which investigation remains under-represented in contrast to the plethora of studies dealing with interference. Our aim is to study the time-course of semantic *facilitation* in picture naming, using a picture-word “interference” paradigm and event-related potentials (ERPs).

**Methods**: We compared two different types of semantic relationships, associative and categorical, in a single word priming and a double word priming paradigm. The primes were presented visually with a long negative Stimulus Onset Asynchrony (SOA), which is expected to cause facilitation.

**Results**: Shorter naming latencies were observed after both associative and categorical primes, as compared to unrelated primes, and even shorter latencies after two primes. Electrophysiological results showed relatively late modulations of waveform amplitudes for both types of primes (beginning ~330 ms post picture onset with a single prime and ~275 ms post picture onset with two primes), corresponding to a shift in latency of similar topographic maps across conditions.

**Conclusion**: The present results are in favor of a post-lexical locus of semantic facilitation for associative and categorical priming in picture naming and confirm that semantic facilitation is as relevant as semantic interference to inform on word production. The post-lexical locus argued here might be related to self-monitoting or/and to modulations at the level of word-form planning, without excluding the participation of strategic processes.

## Introduction

In everyday conversations, we choose effortlessly the right words in our vast mental lexicon to communicate the meaning we intend to. Most serial models of speech production consider that the spread of activation from conceptual (pre-lexical) representations to lexical selection is semantically driven. In that context, lexical selection is seen as a decisional process, during which we have to select the right “target” word among other semantically related “non-target” words. These co-activated non-target words can either create a facilitatory or an inhibitory context, respectively speeding up or slowing down speech planning (Roelofs, 1992, 2006; Chen and Mirman, 2012). In language production research, semantic interference effects (i.e., word production slowed down by an inhibitory semantic context) have received a lot of interest. They have been mainly studied with the Stroop task (for an integrative review, see MacLeod, 1991) and its variant, the picture-word interference paradigm (PWIP), in which pictures instead of colors are used and the asynchrony of the picture-word pair presentation is manipulated (for the first description of a PWIP, see Rosinski et al., 1975). Contrasting with the plethora of studies dealing with interference, only a few investigations adressed semantic facilitation effects (for a discussion, see Mahon et al., 2007); and yet, “natural” semantic contexts seem to be generally facilitative rather than interfering, as it is usually shown in semantic categorization tasks (Kuipers and La Heij, 2008; Hantsch et al., 2012), semantic priming tasks in speech perception (Lucas, 2000) and picture naming after a constraining sentential context (Griffin and Bock, 1998; Piai et al., 2014b, 2015). Psycholinguistic studies may actually have focused too much on interfering semantic effects, i.e., on the exception that proves the rule. In that sense, Mahon et al. (2007, p. 505) argued “*that the critical data that should inform models of lexical selection are semantic facilitation effects*” and not (only) interference effects.

In the PWIP, semantic interference and facilitation effects are defined as a difference of mean latencies when naming a picture in two experimental conditions: a semantic-high condition (usually a word from the same category as the target picture) and a semantic-low condition (a word unrelated with the target picture). Semantic interference in the PWIP has been typically obtained with a semantic-high condition consisting of coordinates (e.g., pear-apple; Rosinski et al., 1975; Rosinski, 1977; Lupker, 1979; La Heij, 1988; La Heij and van den Hof, 1995; Starreveld and La Heij, 1996; Vitkovitch and Tyrrell, 1999; Costa et al., 2005; Finkbeiner and Caramazza, 2006; Sailor et al., 2009; Damian and Spalek, 2014), independently of the strength of the categorical overlap between the picture and the word (Hutson and Damian, 2014). But semantic interference in the PWIP has also been obtained with subordinates (e.g., tulip-flower; Hantsch et al., 2005, 2012), superordinates (e.g., bird-seagull; Hantsch et al., 2005; Kuipers et al., 2006) and part-terms (e.g., window-car; Sailor and Brooks, 2014). Even if the typical and often described result is interference, the PWIP can just as much induce semantic facilitation, yet under certain experimental conditions. Semantic facilitation in the PWIP has been obtained with a large panel of semantically related words: coordinates (Finkbeiner and Caramazza, 2006), subordinates (Costa et al., 2003), superordinates (Vitkovitch and Tyrrell, 1999; Damian and Abdel Rahman, 2003), associates not being coordinates (e.g., milk-cow; Alario et al., 2000; Costa et al., 2005; Sailor et al., 2009; de Zubicaray et al., 2013; Damian and Spalek, 2014; Sailor and Brooks, 2014), related adjectives (e.g., chilly-ice; Bölte et al., 2013) and related verbs (e.g., sit-chair; Mahon et al., 2007). More crucially, some PWIP studies demonstrated that the exact same set of materials used in slighlty different experimental settings can shift the polarity of the effect from interference to facilitation. For instance, the Stimulus Onset Asynchrony (SOA) seems to play a predominant role: several studies (Glaser and Düngelhoff, 1984; Alario, 2001; Bloem et al., 2004; Zhang et al., 2016) reported semantic interference particularly with near-to-0 SOAs but semantic facilitation with long negative SOAs (from −400 ms to −1000 ms). The polarity of the effect can also be affected by the visibility of the primes (Finkbeiner and Caramazza, 2006), the modality of the primes and the rate of congruent responses (Hantsch et al., 2009), and the presence of a familiarization phase (Collina et al., 2013).

The large amount of studies on semantic interference has given rise to different interpretative hypotheses regarding its underlying mechanisms and served to develop models of lexical access. Interfering effects in the PWIP have often been interpreted as reflecting the competition between lexical representations (Schriefers et al., 1990; Levelt et al., 1999; Damian and Bowers, 2003; Howard et al., 2006). Due to lexical competition, the latency of the target word selection “mathematically” depends on the state of activation of the non-target words (Levelt et al., 1999). In other words, it takes longer to select the right lexical representation among a high number of competitors strongly co-activated. However, other models locate the competition process at a pre-lexical stage, via a learning mechanism weakening the semantic-to-lexical connections (Oppenheim et al., 2010) or at a post-lexical stage (Finkbeiner and Caramazza, 2006; Mahon et al., 2007; Janssen et al., 2008). The response-competition (or response-exclusion) hypothesis indeed states that in presence of a picture-word pair, the distractor word is automatically encoded and put in the phonological output buffer and ready to be produced (Lupker, 1979; Roelofs, 2003). The distractor word has to be detected and deleted before being able to produce the target word associated to the picture. The more semantic features are shared between the picture and the interfering word, the more time will be necessary to remove the distractor word from the response buffer. For example, the picture “cat” associated with the word “dog” leads to a very competitive situation, because the overlap of semantic features is high, making the dog a relevant candidate when naming a cat. Consequently, the exclusion of a semantically close potential candidate increases naming latencies in the PWIP. Finally, some authors argue that a general mechanism responsible for checking online the accuracy of speech production, the verbal self-monitoring, becomes more alert in semantic-high conditions, either to suppress the buffered response (Dhooge and Hartsuiker, 2012) or to validate the selection of the target-word in the context of highly activated competitors (Maess et al., 2002; Ganushchak and Schiller, 2008).

As for semantic facilitation effects in the PWIP, they have usually been interpreted as reflecting automatic spreading-activation from semantically related conceptual representations to their corresponding lexical representations. According to this view, a higher level of activation of the semantic nodes results in faster lexical selection, independently of the non-target words, i.e., without lexical competition (Dell and O’Seaghdha, 1991; Mahon et al., 2007). Similar to semantic interference, other interpretations do not locate the spreading process at the lexical level. First, the Conceptual Selection Model (Bloem et al., 2004) claims that the co-activated conceptual representations do not automatically activate their corresponding lexical representations. Therefore, the semantic spreading-activation is restricted to the conceptual/pre-lexical level (Collins and Loftus, 1975), leading to an earlier start of the lexical stage and shorter naming latencies. Second, Starreveld and La Heij (1995, 1996) proposed an interactive activation model accounting for context effects in the PWIP, where semantic and phonological levels are bidirectionnally interconnected, and in which semantic similarity effects are located at the post-lexical level. In this connectionist model, the phonological node of the target receives activation both from the picture and the related word due to the connections at the semantic level.

Although relying on different theoretical backgrounds, semantic interference and facilitation in the PWIP have both been alternately interpreted as pre-lexical, lexical or post-lexical effects. These interpretations have been based mostly on behavioral/offline measures (latencies in the case of picture naming), which might not be precise enough to understand which processing stage is affected. For this purpose, event-related potentials (ERPs) have been combined with PWIPs in a few studies to detail the time-course of semantic context effects on single word production. Semantic interference effects were investigated in the framework of lexical competition that seems amplified in the PWIP with near-to-0 ms SOAs. ERP modulations for coordinates (vs. unrelated distractors) were reported starting 230–275 ms (Aristei et al., 2011; Piai et al., 2012; Wong et al., 2017), or 320–350 ms post picture onset (Dell’Acqua et al., 2010; Piai et al., 2014a; Shitova et al., 2016). The 100 ms discrepancy in the time-windows associated to semantic interference in these studies are hardly compatible with a unique interpretation, all the more that the later time-window (320–350 ms after picture onset) falls much beyond estimates situating lexical selection before 275 ms (Indefrey, 2011). Two other studies reported semantic interference for coordinates that was rather interpreted as semantic priming, but again effects on ERPs were observed in very different time-windows: either very early, i.e., 106 ms post picture onset (Dell’Acqua et al., 2010) or quite late, i.e., 325 ms post picture onset (Blackford et al., 2012). Blackford et al. (2012) provided also an alternative interpretation for their late semantic ERP modulations, namely that they possibly mirrored “activity at the phonological word-form representation”. Finally, ERP studies addressing semantic facilitation effects in the PWIP focused on associates: ERP modulations by semantic context started as early as 120 ms post picture onset (Hirschfeld et al., 2008) or around 200 ms post picture onset (Aristei et al., 2011), and were interpreted respectively as reflecting a speed-up of object identification or (pre-)lexical processes.

The aforementioned ERP studies investigating the time-course of semantic interference and facilitation in the PWIP reported discrepant results on their time-course and brought forth different interpretations. As summerized above for the behavioral results, some of these discrepancies may be due to the SOA and to the different types of semantic relationships between the word and the picture. To try to shed light on these two variables, the word-picture relationship (associative vs. categorical) was manipulated in the ERP study reported here. We used a long negative SOA to increase the probability of obtaining semantic facilitation effects (Glaser and Düngelhoff, 1984; Alario, 2001; Bloem et al., 2004; Zhang et al., 2016), on a material previously tested with a short negative SOA (see below in the “Materials and Methods” section).

Among the set of variables influencing semantic effects in the PWIP, the number of words presented with the picture has been shown to increase semantic interference. When two categorical words were presented alongside the picture, they slowed down picture naming (Abdel Rahman and Melinger, 2008), which was interpreted as increased competition due to a higher number of activated lexical competitors. To our knowledge, the presentation of two semantically related words in a PWIP has been used only to study semantic interference (Abdel Rahman and Melinger, 2008; Melinger and Abdel Rahman, 2013), but not facilitation. To address this issue, we compared naming responses and their neural correlates when a picture was preceded by one word or by two words (single vs. double priming hereafter). This manipulation of the amount of semantic contextual priming, as well as the type of priming words (associative/categorical) should make a decisive insight to current models of speech production. The present investigation might offer innovative knowledge about word production: semantic facilitation is inherent in everyday conversation, as we always benefit from a given semantic context to produce speech easily. This follows the argument that semantic facilitation in the PWIP (but also in the blocked-cyclic naming paradigm) could be even *more* natural and *more* relevant than semantic interference to inform on lexical selection (Mahon et al., 2007; Navarrete et al., 2014).

We predicted that participants would be faster when naming pictures after semantically related primes (associative or categorical), as compared to unrelated primes with long negative SOAs. Early ERP modulations around the P2/N2 component (~200–250 ms post picture onset) would presumably point to a lexical locus of semantic facilitation, whereas earlier/later modulations to pre-/post-lexical processes respectively. Multiple words should produce more facilitation than a single word if they boost the activation of the target by overcoming lexical competition, or less facilitation if they increase lexical competition in keeping multiple competitors highly active.

## Materials and Methods

### Participants

Twenty-four French-speaking and right-handed adults participated in this study (aged 19–24, mean 21.1, one male). They were undergraduate students at the University of Geneva and received course credit for their participation. They all had normal or corrected-to-normal vision. None of them had a significant history of neurological disorder. This study was carried out in accordance with the recommendations of the ethical committee of the Faculty of Psychology and Educational Science of Geneva University for research on healthy subjects (“Etude psycholinguistique de la production et compréhension du langage: approches comportementales et électrophysiologiques”) with written informed consent from all subjects in accordance with the Declaration of Helsinki. The protocol was approved by the ethical committee on May 16th 2013.

### Material

A set of 59 black and white line drawings (resized to 240 × 245 pixels) were chosen from two databases (Alario and Ferrand, 1999; Bonin et al., 2003). All pictures had a high name agreement in french (above 70%) and covered 17 different semantic categories (1–13 items per category): food, animals, housekeeping material, trees, weapons, jewels, living places, music instruments, desk material, media, vehicles, tools, body parts, recipients, personal care items, kitchenware, clothes. Each picture/word in this set of 59 stimuli was linked with six words: two associative words, respectively one high-associative word (i.e., more than 14.6% of the subjects gave this word as the first associate in Ferrand and Alario, 1998) and one low-associative word (i.e., less than 14.6% in Ferrand and Alario, 1998), two words belonging to the same semantic category (as stated in Bueno and Megherbi, 2009, when semantic category was available) and two unrelated words. Unrelated words corresponded to half of the associative words and half of the categorical words re-paired to match unrelated targets. According to the database Lexique (New et al., 2004), the associative (ASS), categorical (CAT) and unrelated (UNR) words lists were comparable in terms of lexical frequency and length (phonemes/syllables). None of the associative words belonged to the same semantic category as the target to name and all verbs were changed into their derivated common nouns (e.g., to fly—flight). The priming words did not share the same initial or final phonemes with the target to name. The high-associative word or the most frequent categorical word was presented in case of single priming and in first position in case of double priming. An item example is given in Table 1 and the entire list of word stimuli can be found in the Supplementary Material.

**Table 1:** Example of prime samples for the target picture “airplane”.

	Associative	Categorical	Unrelated
Single priming	Flight	Helicopter	Rope
Double priming	Flight, sky	Helicopter, bus	Rope, shovel

In total, each target picture appeared six times throughout the experiment, i.e., in three different conditions (ASS, CAT, UNR) and preceded by one or two words (single or double priming). The stimuli presentation order was pseudo-randomized and counterbalanced in twelve different lists, so that the same target pictures were separated by at least 20 other pictures and the condition (ASS, CAT, UNR) was identical for a maximum of two consecutive trials. In each list, the number of primes that preceded the target (i.e., one or two) was randomly mixed.

The material was tested in a preliminary study in which a written single word was presented 66 ms before the picture to name, in order to assess if it was able to elicit the classical semantic interference effect (at least with coordinates). After a fixation cross (1250 ms onscreen), the word prime was presented for 53 ms, and a 13 ms blank screen preceded the picture (SOA −66 ms) which remained 2000 ms on screen. The inter-stimulus interval lasted 1000 ms. All other manipulations and behavioral data analyses were the same as in the ERP experiment (see below). Twenty-eight undergraduate students took part in this preliminary study, and did not take part in the ERP experiment (aged 18–35, five males).

As expected, categorical semantic interference was observed with slower reaction times with categorical primes (mean RT 806 ms) relative to unrelated primes (mean RT 791 ms; *t*_(4623)_ = 3.499, *p* < 0.001) and to associative primes (mean RT 785 ms; *t*_(4620)_ = 4.675, *p* < 0.001), but no difference was found between associative and unrelated primes (*t*_(4622)_ = 1.185, *p* = 0.24).

### Procedure

Participants sat in a comfortable desk chair in a sound-proof room, approximately 50 cm in front of a computer screen. They were randomly assigned to one of the 12 counterbalanced lists. Before the experiment, they first underwent a familiarization phase consisting in reading aloud the names written underneath the 59 target pictures. Then, they performed a practice phase consisting in naming the 59 target pictures once, without display of the names. This double pre-exposure was supposed not only to avoid naming errors and hesitations, but also to minimize the repetition priming and the role of object identification during the experiment (Francis, 2014). In these two early phases, they could proceed at their own pace, the pictures being presented one by one on the computer screen in alphabetical order.

As for the task itself, the subjects were instructed to name the pictures as quickly and accurately as possible and there were three warming-up filler trials before beginning the experiment. The trials were presented through E-Prime 2.0 software^1^ (Psychology Software Tools, Pittsburgh, PA, USA) and responses were recorded with an external microphone.

On each trial, a green fixation plus sign (+) appeared in the center of the screen for 250 ms. The plus sign remained on screen until the presentation of the written prime(s): a written word prime was presented in the center of the screen in font Courier New 48 for 554 ms (SOA −700 ms). In case of double priming, the second word prime was presented alike after a blank screen of 150 ms (SOA −1400 ms). A blank screen was then presented for 150 ms and finally the picture appeared on the center of the screen for 2000 ms. Between each trial, another blank screen was presented during 2000 ms. Subjects were encouraged to blink in this time period. There was a break after each quarter of the experiment, which lasted approximately 35 min in total.

The electroencephalogram (EEG) was acquired by the continuous recording of 128 electrodes placed on a soft nylon cap with standard 10-5 locations (Oostenveld and Praamstra, 2001). Signals were recorded with the Biosemi ActiveTwo system (Biosemi V.O.F Amsterdam, Netherlands) at a 512 Hz sample, with filters DC to 104 Hz and 3 dB/octave slope.

### Data Analysis

#### Behavioral Analysis

We first excluded from the behavioral data all trials in which participants did not produce the expected single target word or when no response was given within the 2 s time limit. For every correct trial, the reaction time (RT) corresponding to the vocal onset was defined manually according to the spectrogram and the waveform with the software Check Vocal (CheckVocal 2.2.6, Protopapas, 2007). Naming latencies situated below or above three standard deviations of each subject’s mean were excluded. The statistical analyses were conducted with R software (R Development Core Team, 2003). The errors were analyzed with generalized mixed-effects models for binomial distributions (Jaeger, 2008) and the production latencies with linear mixed-effects regression models (Baayen et al., 2008).

#### EEG Analysis

The pre-analyses were conducted with Cartool software 3.60 (Brunet et al., 2011). Epochs of 600 ms time-locked to 150 ms before the picture onset (stimulus-locked) and epochs of 450 ms time-locked to 100 ms before the vocal onset (response-locked) were extracted and averaged for each subject across conditions, with butterworth filters set to 0.2–30 Hz (2nd order acausal Butterworth filter with −12 dB/octave roll-off). All epochs related to correct productions were recalculated against the average reference, visually inspected and accepted only in the absence of artifact, such as eyeblinks, motor artifacts or large amplitude variations. Only trials with artifact-free stimulus- and response-locked epochs were retained. Contaminated electrodes (up to 15% of the 128 electrodes) were interpolated with a 3-D splines interpolation (Perrin et al., 1987).

The FieldTrip MATLAB software toolbox (Oostenveld et al., 2011) with custom scripts was used to analyze the waveform amplitudes over the entire data set at each time point on the 128 electrodes separately on stimulus- and response-locked ERPs. In order to identify in which time-windows significant clusters showed divergences in ERP amplitudes between the unrelated condition (UNR) and the semantically related conditions (ASS, CAT), non-parametric cluster-based permutations were computed (2000 randomizations with a spatial threshold at four clustered electrodes and alpha criterion above 0.01 for each time point). We used such a conservative threshold in order to minimize the bias of multiple comparisons. In the spatio-temporal/topographic analysis, we first tested whether conditions showed significant differences in global dissimilarity using non-parametric randomization tests (called “TANOVAs” without being an analysis of variance) with the RAGU software (Koenig et al., 2011). Data were normalized (L2 norm) and 5000 runs of randomization were computed. “TANOVAs” were calculated between the unrelated condition (UNR) and each of the semantically related conditions (ASS, CAT). Topographic differences in time-windows longer than 20 ms and with an alpha criterion below 0.01 for each time point were retained. In order to cover the entire planning process in each condition, the grand means of stimulus- and response-locked epochs were combined according to RTs by removing the overlapping signal. The duration of the combined waveforms of the grand averages corresponded to the mean naming latencies of the group of subjects in each condition and the same procedure was applied to each individual ERP. Then, the spatio-temporal clustering of stable microstate maps was conducted on the grand averages with the K-Means clustering algorithm in the Cartool software (5000 runs of randomization). This procedure segments ERPs in periods of quasi-stable global electrophysiological patterns at scalp (i.e., topographic maps or ERP microstates) by compressing the variability of ERPs in a series of template maps which summarize the data according to which topographic template best explains the group-averaged ERP responses to each experimental condition (Pascual-Marqui et al., 1995; Michel and Murray, 2012). The selection of the optimal number of ERP maps that best explain the group-averaged data across conditions was based on a combination of multiple criteria such as cross-validation and Krzanovski-Lai (see Murray et al., 2008). Statistical smoothing was applied to remove temporally isolated topographic maps with low explanatory power. Clusters that correlated above 97% were merged and segments shorter than 20 ms were rejected. The statistical validation of this analysis was obtained with a fitting procedure that consisted in comparing each of the microstates observed in the grand averages with the moment-by-moment scalp topography of single-subjects’ ERPs. Repeated measures analysis of variance (ANOVAs) were then calculated with an alpha criterion below 0.01 to compare the mean duration and the mean global explained variance (GEV) of the fitted microstates across subjects in each condition, with Fisher LSD tests for the pairwise comparisons.

## Results

### Behavioral Results

Of the 8496 trials, 1.94% were errors and 1.79% were situated below or above three standard deviations of each subject’s mean, leading to 3.73% data loss. The errors were distributed as follows: categorical semantic (41%), no responses (22%), hesitations (21%), unrelated (5%), associative semantic (4%), phonological (4%) and morphological (3%). In the error analysis, the ASS condition led to a lower rate of errors, as compared to the UNR (*z* = 1.923, *p* = 0.05) and the CAT conditions (*z* = −2.014, *p* = 0.04), but there was no difference between UNR and CAT conditions (*z* = −0.094, *p* = 0.925).

The production latencies for each condition are presented in Table 2. The analyses revealed a significant effect of condition (*F*_(2,8083.9)_ = 60.323, *p* < 0.001), number of primes (*F*_(1,8083.8)_ = 68.76, *p* < 0.001), item repetition (*F*_(5,8083.9)_ = 50.264, *p* < 0.001) and a significant interaction between the condition and number of primes (*F*_(2,8083.8)_ = 7.676, *p* < 0.001). We therefore conducted separate analyses for single and double priming.

**Table 2:** Mean reaction time (and standard deviation) in ms for each condition.

	Associative	Categorical	Unrelated
Single priming	697 (154)	706 (146)	720 (141)
Double priming	662 (161)	688 (138)	709 (142)

In single word priming, the model indicated a significant effect of condition (*F*_(2,3984)_ = 13.831, *p* < 0.001) and item repetition (*F*_(5,3984)_ = 20.541, *p* < 0.001). Pairwise comparisons showed that both CAT and ASS conditions led to faster RTs as compared to UNR condition (CAT vs. UNR : *t*_(3985)_ = −3.281, *β* = −15.34, SE = 4.68, *p* = 0.001; ASS vs. UNR : *t*_(3985)_ = 5.137, *β* = −24.02, SE = 4.68, *p* < 0.001). Although only marginally significant (*t*_(3985)_ = −1.857, *β* = 8.68, SE = 4.68, *p* = 0.06), the ASS condition also led to faster RTs as compared to the CAT condition. In double word priming, significant effects of condition (*F*_(2,4013)_ = 53.391, *p* < 0.001) and item repetition (*F*_(5,4013)_ = 30.84, *p* < 0.001) also appeared. A similar pattern to single priming was observed in two by two comparisons of double priming (ASS vs. CAT: *t*_(4014)_ = 5.887, *β* = 28.17, SE = 4.79, *p* < 0.001; CAT vs. UNR : *t*_(4014)_ = −4.193, *β* = −20.14, SE = 4.8, *p* < 0.001; ASS vs. UNR : *t*_(4013)_ = −10.113, *β* = −48.3, SE = 4.78, *p* < 0.001).

In summary, RTs decreased as follows through the conditions: UNR > CAT > ASS, with a further speeding effect of the double word priming.

### EEG Results

All 24 subjects were included in the subsequent analysis with 61%–98.3% of artifact-free epochs accepted per condition. We conducted separate analyses for single priming and double priming, due to the interaction between the condition and the number of primes.

#### Single Word Priming

**Figure 1:**
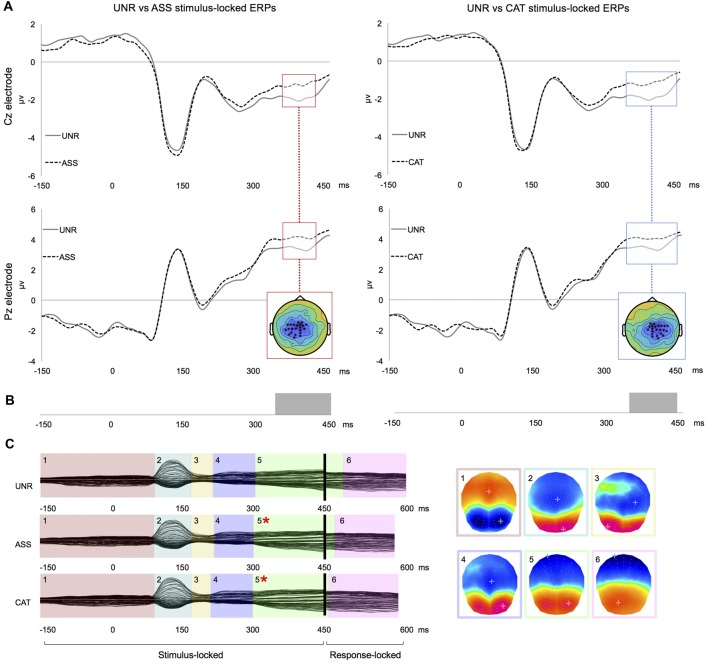
Results for single word priming. **(A)** Examples of group-averaged waveforms for the UNR-ASS (left) and UNR-CAT (right) contrasts on stimulus-locked ERPs: time-windows of significant clusters over at least four electrodes are highlighted and all electrodes showing the highlighted effect are crossed on the topographical representation. **(B)** Results of “TANOVA” spatio-temporal analysis for the same contrasts: bars represent time-periods of significant differences in global similarity. **(C)** Temporal distribution of stable electrophysiological patterns at scalp from the spatio-temporal segmentation on the combined stimulus- and response-locked grand averages matching the actual reaction times of each experimental condition (*indicates a significant difference in map duration).

In the waveforms’ amplitudes analysis of stimulus-locked ERPs, there were significant differences from about 355 to 420 ms post-picture onset between the UNR and ASS conditions (Figure 1A left), and from about 330 to 430 ms post-picture onset between the UNR and CAT conditions (Figure 1A right). In both cases, the differences concerned large clusters of central electrodes. In this time-window, the waveforms of the related conditions (CAT and ASS) were less negative than the unrelated condition notably on the electrode Cz (Figure 1A). There were no significant amplitude differences between ASS and CAT conditions on stimulus-locked ERPs. No difference was observed on response-aligned ERPs whenever.

In the spatio-temporal segmentation analysis, a topographic consistency test (Koenig and Melie-García, 2010) confirmed a consistent pattern of active sources for each condition across subjects during the whole stimulus- and response-locked averaged epochs. The TANOVAs revealed topographic differences from about 335 to 450 ms post picture onset for the UNR-ASS comparison (Figure 1B left), and from about 340 to 435 ms post picture onset for the UNR-CAT comparison (Figure 1B right), closely matching the time-windows of significant differences on waveforms’ amplitudes. TANOVAs between ASS and CAT conditions revealed no significant topographic difference. In response-locked ERPs, no topographic difference appeared between conditions. The spatio-temporal segmentation on the combined grand average ERPs starting 150 ms pre-picture to 100 ms pre-response identified six different periods of quasi-stable topographic patterns summarizing the EEG signal of each condition and accounting for 98.1% of the variance of the data (Figure 1C). Following visual inspection of the time distribution of those six maps and the TANOVAs results, the fitting of these topographic patterns in the individual ERPs was conducted in two different time-windows: 0–300 ms (first four map templates) and 300 ms to the end of the signal (last two maps). The mean duration and GEV of each map per condition across participants are presented in Table 3. The GEV corresponds to the variance within these two fitting time-windows (and not within the entire planning period). Statistical comparisons on the duration of each map between conditions were significant only for map 5 (*F*_(2,46)_ = 6.899, *p* = 0.002). Map 5 was shorter in the ASS (*p* = 0.003) and CAT conditions (*p* = 0.002) as compared to the UNR condition. The GEV analysis showed convergent results as the GEV was modulated by the conditions only in map 5 (*F*_(2,46)_ = 5.268, *p* = 0.009). The GEV was higher in the ASS (*p* = 0.02) and CAT (*p* = 0.004) conditions as compared to the UNR condition.

**Table 3:** Mean duration (in ms) and Global Explained Variance (GEV, in %) of the six microstates in each condition for single word priming according to the fitting procedure in the individual ERPs.

		Fitting from 150 ms before picture to 300 ms				Fitting from 300 ms to 100 ms before RT	
		Map 1	Map 2	Map 3	Map 4	Map 5	Map 6
UNR	*Duration (ms)*	249	117	26	58	166	154
	*GEV (%)*	9.5%	5.9%	1.0%	2.3%	13.7%	12.0%
ASS	*Duration (ms)*	243	110	35	64	132	164
	*GEV (%)*	9.2%	5.8%	1.1%	2.8%	11.5%	12.5%
CAT	*Duration (ms)*	244	128	27	52	130	173
	*GEV (%)*	9.5%	6.3%	0.9%	2.3%	10.9%	14.0%

#### Double Word Priming

**Figure 2:**
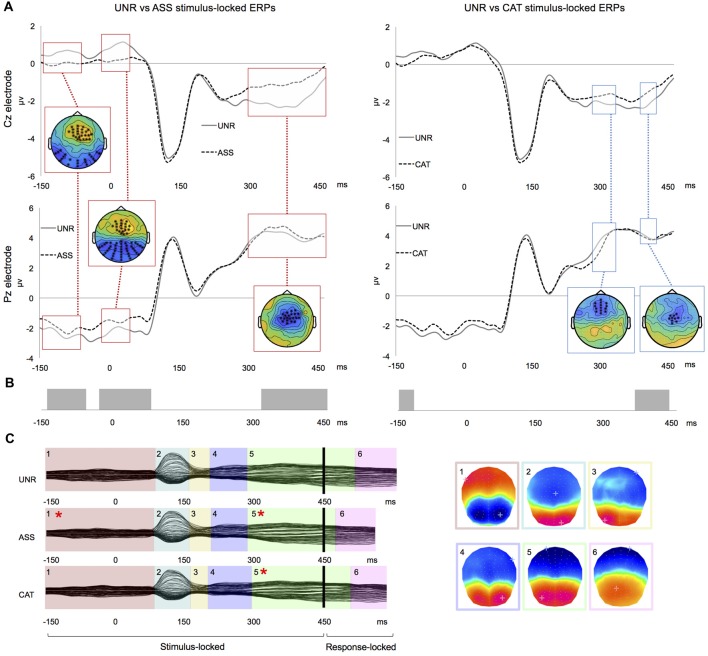
Results for double word priming. **(A)** Examples of group-averaged waveforms for the UNR-ASS (left) and UNR-CAT (right) contrasts on stimulus-locked ERPs: time-windows of significant clusters over at least four electrodes are highlighted and all electrodes showing the highlighted effect are crossed on the topographical representations. **(B)** Results of “TANOVA” spatio-temporal analysis for the same contrasts: bars represent time-periods of significant differences in global similarity. **(C)** Temporal distribution of stable electrophysiological patterns at scalp from the spatio-temporal segmentation on the combined stimulus- and response-locked grand averages matching the actual reaction times of each experimental condition (*indicates a significant difference in map duration).

In the waveforms’ amplitudes analysis, the UNR-ASS comparison showed significant differences in three time-windows: from −145 ms to −70 ms pre-picture, from −30 ms pre-picture to 35 ms post-picture onset and from 285 ms to 445 ms post-picture onset (Figure 2A left). In the first and second time-windows, the differences involved large clusters of anterior and posterior electrodes, whereas in the third time-window, they were on central electrodes. In the UNR-CAT comparison, two time-windows of significant amplitude differences emerged: 275–320 and 375–405 ms post-picture onset (Figure 2A right), involving clusters of anterior and central electrodes. The waveforms of the related conditions were less negative than the unrelated condition (Figure 2A). The ASS-CAT comparison of stimulus-locked ERPs showed significant amplitude differences from −35 ms pre-picture to 25 ms post-picture onset. There were no significant amplitude differences in the response-locked ERPs.

In the spatio-temporal segmentation analysis, a topographic consistency test (Koenig and Melie-García, 2010) also confirmed a consistent pattern of active sources for each condition across subjects during the whole stimulus- and response-locked averaged epochs. The UNR-ASS comparison revealed significant topographic differences −140 to −60 ms pre-picture, −30 ms pre-picture to 85 ms post picture onset and from 310 to 450 ms post picture onset (Figure 2B). The UNR-CAT comparison showed topographic differences −140 to −115 ms pre-picture and from 360 ms to 430 ms post-picture onset (Figure 2B). As for the ASS-CAT comparison, it revealed topographic differences in two time-windows, namely −35 ms pre-picture to 35 ms post-picture onset and 55–80 ms post-picture onset, overlapping waveform amplitudes differences (not illustrated). In response-locked ERPs, no significant topographic difference appeared between the conditions.

The spatio-temporal segmentation on the combined ERP grand averages and the fitting in the individual ERPs was conducted in the same way as for single priming (Figure 2C). Again, six different microstates were found, accounting for 97.9% of the variance of the data (Figure 1C). Given the similar distribution of maps, the same fitting periods as for single word priming were used. When comparing the duration of each map per condition (Table 4), significant differences were found on map 1 (*F*_(2,46)_ = 5.305, *p* = 0.008) and map 5 (*F*_(2,46)_ = 10.831, *p* < 0.001). More precisely, map 1 was shorter in the ASS condition as compared to the two other conditions (ASS vs. UNR: *p* = 0.002; ASS vs. CAT: *p* = 0.05), without duration difference between UNR and CAT (*p* = 0.25). As for map 5, it was significantly shorter in semantic-high conditions as compared to the semantic-low condition (UNR vs. ASS: *p* < 0.001; UNR vs. CAT: *p* = 0.008), without significant difference between ASS and CAT (*p* = 0.07). For both maps, convergent results were found on the GEV, which varied significantly across conditions for map 1 (*F*_(2,46)_ = 13.538, *p* < 0.001) and map 5 (*F*_(2,46)_ = 10.603). On map 1, the GEV was lower in the ASS condition as compared to the UNR (*p* < 0.001) and CAT (*p* < 0.001) conditions, with no difference between UNR and CAT (*p* = 0.24). On map 5, the GEV in the semantic-high conditions was lower than in the UNR condition (UNR vs. ASS: *p* < 0.001; UNR vs. CAT: *p* = 0.006), but no difference appeared between ASS and CAT (*p* = 0.09).

**Table 4:** Mean duration (in ms) and Global Explained Variance (GEV, in %) of the six microstates in each condition for double word priming according to the fitting procedure in the individual ERPs.

		Fitting from 150 ms before picture to 300 ms				Fitting from 300 ms to 100 ms before RT	
		Map 1	Map 2	Map 3	Map 4	Map 5	Map 6
UNR	*Duration (ms)*	246	90	30	86	194	114
	*GEV (%)*	10.6%	4.7%	1.1%	4.2%	18.3%	9.7%
ASS	*Duration (ms)*	212	122	32	85	134	131
	*GEV (%)*	8.3%	5.7%	1.1%	4.2%	12.4%	10.7%
CAT	*Duration (ms)*	233	104	33	81	158	128
	*GEV (%)*	10.0%	5.2%	1.0%	3.9%	14.6%	10.8%

## Discussion

Using a PWIP with a rather long negative SOA, we observed that picture naming was speeded up with associative and categorical primes, as compared to unrelated primes. A lengthened SOA was sufficient to overcome the interfering effect that was observed in the preliminary experiment at a SOA of −66 ms. Interestingly, associative primes accelerated the speech planning process more than categorical primes did. This is in line with previous reports showing that thematic/associative relations induce less lexical competition than categorical ones (e.g., Costa et al., 2005). When the prime and the target belong to the same category (e.g., penguin-eagle), they also share several semantic features, leading to the activation of multiple concepts spreading to multiple lexical entries competing for selection. But when the prime and the target are associated (e.g., penguin-ice), less semantic features are shared and lexical competition is weaker (Abdel Rahman and Melinger, 2009) or absent (de Zubicaray et al., 2014), as compared to the categorical condition. In addition, picture naming was also faster with a double priming as compared to a single priming in the ASS and CAT conditions. In the same way as in semantic interference (Abdel Rahman and Melinger, 2008), we provide here the first observation that multiple primes can increase semantic facilitation effects. However, contrary to interference effects, the present result can hardly be explained by increased lexical competition. In the next sections, we will discuss the time-windows of the semantic facilitation effects and how they contribute to the understanding of the processes underlying speech production.

### Time-Windows of Semantic Facilitation

When comparing semantic-high conditions (ASS, CAT) with the semantic-low condition (UNR), ERP analyses showed consistent amplitude modulations in relatively late time-windows in stimulus-locked ERPs, i.e., beyond 330 ms post picture onset in single priming and beyond 275 ms in double priming, with larger amplitudes for ASS and CAT relative to UNR. Despite a 50 ms shift in the onset of ERP effects between single and double priming, both fall beyond the P2/N2 component which has been previously associated with the onset of lexical selection. This shift might therefore only reflect the overall speed difference (mean RT for single priming: 708 ms and for double priming: 686 ms). This late locus is confirmed by the spatio-temporal segmentation analyses: duration differences appeared systematically on the microstate starting around 300 ms post picture onset (map 5 in Figures 1C, 2C), which was shorter in semantic-high conditions. Therefore, the observed ERP results likely correspond to shorter duration of similar mental processes for the primed conditions in a time-window falling after lexical selection.

In double priming, ERP amplitude modulations between the UNR and ASS conditions began already in the blank interval between the words and the picture, with larger amplitudes for UNR relative to ASS. These results converge with those from the ERP spatio-temporal analyses, in which the first microstate (map 1 in Figure 2C) is shorter in the ASS condition than in the other conditions. The pre-picture ERP effects could be due to an integration of the link between the two associative primes leading to lexical preactivation/anticipation (Dikker and Pylkkänen, 2013). Interestingly, no early/pre-picture amplitude modulation was found when comparing UNR and CAT conditions, whereas ASS vs. CAT divergences also showed up around the picture onset. Indeed, with two ASS primes (e.g., flight, sky), the target (e.g., airplane) that underwent familiarization was quite predictable, whereas after two CAT primes (e.g., helicopter, bus), only the semantic category could be anticipated and the target remained less predictable. Some subjects could have tried to apply a strategy, i.e., guessing or anticipating each naming response during or quickly after the presentation of the associative primes. Even if this could partly account for the observed facilitation, we do not believe that our results are only due to pure guessing for the following reasons. First, semantic facilitation has been reported even with short negative SOAs (Alario et al., 2000; Damian and Abdel Rahman, 2003; Finkbeiner and Caramazza, 2006; Bölte et al., 2013; Damian and Spalek, 2014) and is not proportional to the negative SOA duration (i.e., the amount of facilitation is the same at −1000 ms and −400 ms SOAs; Zhang et al., 2016). Second, clearly anticipated responses (and their corresponding ERPs) were likely eliminated by cleaning the reaction times with a cut-off set at three standard deviations for each subject’s mean. Third, the late ERP effects of condition after two associative primes were distributed on a similar cluster of central electrodes than after one associative prime (Figures 1A, 2A).

As the longer negative SOA in double priming vs. single priming might just as well explain the greater facilitation that we reported here, we cannot draw conclusions about the nature of the cumulative effect of multiple primes. It could be due to strategic anticipation (i.e., the subjects know that after having seen two words the picture will appear, whereas after the first word, it can be another word or the picture) and/or from the integration of both words in relation to the target picture/concept to name. Note that after two categorical primes, the amplitude differences concern first a cluster of anterior electrodes and then central electrodes, which could also be—to some extent—indicative of partial anticipation in presence of two primes. Therefore, we will not extrapolate in comparing directly single to double priming, but rather focus on the effect of the types of primes.

### Underlying Processes of Semantic Facilitation

ERP results across semantic relatedness (ASS/CAT) showed effects in the same relatively late time-window (P3/N3 components), on the same clusters of central electrodes and on same periods of stable electrophysiological pattern at scalp. This convergence of ERP effects might suggest that in the PWIP, at least with a long negative SOA, different semantic relationships between the prime and the target (e.g., coordinate vs. associate) can both facilitate speech production in similar ways. Moreover, it seems that the two types of primes exerted an influence on the same speech planning process in the present experiment.

Crucially, the time-window modulated by the semantic manipulation in our experiment fell beyond the P2/N2 component, which has been related to the onset of lexical selection (Maess et al., 2002; Costa et al., 2009; Strijkers et al., 2010; Aristei et al., 2011). Such late effects of semantic primes in the PWIP have been previously reported by other ERP studies (Dell’Acqua et al., 2010; Blackford et al., 2012; Shitova et al., 2016), although these studies used only coordinates and observed behavioral interference. Shitova et al. (2016) situated the effect at the word-planning stage, and the two other studies (Dell’Acqua et al., 2010; Blackford et al., 2012) provided slightly more specific interpretations and related this late effect to the activation of the phonological word-form of the picture’s name. Indeed, post-lexical processes are very likely engaged after 275 ms: this time-window has been associated in previous ERP studies to phonological processes (Vihla et al., 2006), impaired phonological encoding (Laganaro et al., 2009, 2011) and differed between phonological and orthographical word form encoding in picture naming (Perret and Laganaro, 2012). Critically, “post-lexical” does not necessarily mean phonological or phonetic. It has also been shown that other variables like word age of acquisition, name agreement and image agreement modulate ERPs in late time-windows associated with post-lexical processes (Valente et al., 2014), which is in line with monitoring going on in parallel to word form encoding.

This leaves us with two possible accounts for post-lexical effects: modulations at the word-form level or/and monitoring effects. The self-monitoring internal loop, engaged as soon as the first segment of the phonological word is encoded (Indefrey and Levelt, 2004), might have been more efficient in the semantic-high conditions than in the unrelated condition. Semantic primes could have lowered the resources needed by the monitoring for the phonological preparation. Such a predominant role of self-monitoring in the PWIP is supported by the study of Dhooge and Hartsuiker (2012). According to these authors, only the implication of verbal self-monitoring can account for the polarity reversal of the effects observed in the PWIP. Interestingly, the PWIP study of Dell’Acqua et al. (2010) showed concomitant ERP effects for opposite behavioral effects (phonological facilitation and semantic interference) around 320 ms post picture onset, thus both compatible with the self-monitoring interpretation.

Finally, strategic processes presumably arising post-lexically might also partly explain the present “late” ERP effects: it is possible that participants adopted anticipatory strategies induced by the long negative SOA to predict some naming responses in semantic-high conditions (Alario, 2001). Even if anticipating a response by means of predictions seems very natural in everyday dialogs (Corps et al., 2018) and in sentence completion (Piai et al., 2014b), we cannot affirm straightforwardly that such anticipatory strategies have influenced the (post-lexical) time-course of speech planning and/or the self-monitoring in the present experiments. In language comprehension, prediction and anticipation already drawed the attention of psycholinguists (e.g., Brothers et al., 2015; Luke and Christianson, 2016), but in language production strategic aspects remain under-investigated until now and the level at which predictions are specified is still debated (Drake and Corley, 2015).

In sum, this article demonstrates that semantic facilitation is as suitable as semantic interference to inform on the word production process. It provides the first empirical evidence that categorical and associative facilitation share the same locus, at least in the context of the PWIP with a relatively long negative SOA. It also reports the first observation that multiple primes can increase semantic facilitation. The present data suggest that semantic facilitation effects induced by both ASS and CAT primes arise at post-lexical processing stages, without discarding the involvement of strategic processes. This interpretation is in line with Starreveld and La Heij (2017) recently warranting that Stroop and PWIP both have a “late” locus. By post-lexical, we refer either to phonological processes interacting with (pre-)lexical processes (e.g., semantic integration of the prime with the target during phonological encoding), or to the implication of the verbal self-monitoring. These inferences do not seem to be restricted to the PWIP, as we recently identified ERP facilitation effects also occurring after the P2 component in the first cycle of the blocked cyclic naming paradigm (Python et al., 2018). If we stick to actual estimates for picture naming RT in the 600 ms range (Indefrey, 2011), all effects observed after 275 ms post picture onset are presumably post-lexical. Nonetheless, with mean naming latencies of 700 ms ± 150 ms, the debate remains open about the adequate way of rescaling the actual estimates to various naming latencies (Laganaro, 2016; Roelofs and Shitova, 2017). Future research is needed to better take into account the RT variability in the ERP analysis and identify not only serial stages of speech planing but consider the processes in an interactive way.

## Data Availability Statement

The list of stimuli is included in the supplementary file.

## Author Contributions

GP and ML: study conception and design. GP and RF: acquisition of data. GP, RF and ML: analysis and interpretation of data. GP: drafting of manuscript. RF and ML: critical revision.

## Conflict of Interest Statement

The authors declare that the research was conducted in the absence of any commercial or financial relationships that could be construed as a potential conflict of interest.

## Footnotes

^1^Psychology Software Tools, Inc. [E-Prime 2.0]. Retrieved from http://www.pstnet.com/
