# Supplementary material for: When Wine and Apple Both Help the Production of Grapes: ERP Evidence for Post-lexical Semantic Facilitation in Picture Naming
Source: Front Hum Neurosci. 2018 Apr 10;12:136. doi: 10.3389/fnhum.2018.00136 (PMC5902702; doi:10.3389/fnhum.2018.00136)
Supplement: Supplementary file 1 [file Data_Sheet_1.DOCX]

**Supplementary Material**

| **target** | **associate 1** | **associate 2** | **coordinate 1** | **coordinate 2** | **unrelated 1** | **unrelated 2** |
| --- | --- | --- | --- | --- | --- | --- |
| **araignée**  **(spider)** | toile  (web) | pattes  (legs) | fourmi  (ant) | cafard  (cockroach) | veste  (jacket) | cinéma  (cinema) |
| **autruche**  **(ostrich)** | oeuf  (egg) | plume  (feather) | coq  (rooster) | pigeon  (pigeon) | piano  (piano) | cheveux  (hair) |
| **avion**  **(airplane)** | vol  (flight) | air  (air) | hélicoptère (helicopter) | bus  (bus) | cordes  (ropes) | pioche  (pickaxe) |
| **balai**  **(broom)** | poussière  (dust) | nettoyage  (cleaning) | chiffon  (wipe) | aspirateur (vacuum cleaner) | acteur  (actor) | vent  (wind) |
| **banane**  **(banana)** | singe  (monkey) | peau  (skin) | citron  (lemon) | poire  (pear) | musique  (music) | éléphant  (elephant) |
| **bol**  **(bowl)** | lait  (milk) | déjeuner  (breakfast) | assiette  (plate) | plat  (dish) | jambe  (leg) | chèvre  (goat) |
| **botte**  **(boot)** | pluie  (rain) | caoutchouc  (rubber) | jupe  (skirt) | manteau  (coat) | tigre  (tiger) | vin  (wine) |
| **brouette**  **(wheelbarrow)** | jardin  (garden) | terre  (soil) | seau (bucket) | niveau  (level) | espace  (space) | rat  (rat) |
| **cacahuète**  **(peanut)** | apéritif  (appetizer) | manger  (eat) | noix  (walnut) | figue  (fig) | ailes  (wings) | immeuble  (building) |
| **cactus**  **(cactus)** | désert  (desert) | épine  (needle) | palmier  (palm tree) | sapin  (christmas tree) | toile  (web) | fauteuil  (armchair) |
| **calculatrice**  **(calculator)** | maths  (math) | statistiques (statistics) | papier  (paper) | scotch  (tape) | guitare  (guitar) | montée  (climb) |
| **caméra**  **(video camera)** | cinéma  (cinema) | vidéo  (video) | télévision (television) | micro (microphone) | intelligence  (intelligence) | tente  (tent) |
| **canard**  **(duck)** | mare  (pond) | lac  (lake) | poule  (hen) | dinde  (turkey) | tournevis  (screwdriver) | perle  (pearl) |
| **canon**  **(cannon)** | boulet  (cannonball) | feu  (fire) | fusil  (rifle) | pistolet  (gun) | visage  (face) | ordures  (garbage) |
| **caravane**  **(caravan)** | vacances (vacation) | gitans  (gypsies) | tente  (tent) | villa  (villa) | main  (hand) | tronçonneuse  (chain saw) |
| **carotte**  **(carrot)** | lapin  (rabbit) | poil  (hair) | salade  (salad) | haricot  (bean) | pique  (spine) | mouton  (sheep) |
| **casquette**  **(cap)** | soleil  (sun) | tête  (head) | chapeau  (hat) | pantalon (trousers) | mouche  (fly) | famille  (family) |
| **cerveau**  **(brain)** | intelligence (intelligence) | neurone  (neuron) | oeil  (eye) | coeur  (heart) | poule  (hen) | osier  (wicker) |
| **clown**  **(clown)** | cirque  (circus) | rire  (laugh) | acteur  (actor) | peintre  (painter) | abeille  (bee) | bois  (wood) |
| **coccinelle**  **(ladybird)** | point  (dot) | bonheur (happiness) | mouche  (fly) | guêpe  (wasp) | patin  (ice skate) | ciseaux  (scissors) |
| **collier**  **(necklace)** | perle  (pearl) | diamant  (diamond) | bague  (ring) | chaîne  (chain) | point  (dot) | télévision  (television) |
| **échelle**  **(ladder)** | montée  (climb) | barreaux  (rungs) | pioche  (pick axe) | bidon  (can) | pluie  (rain) | assiette  (plate) |
| **écureuil**  **(squirrel)** | noisette (hazelnut) | forêt  (forest) | rat  (rat) | chien  (dog) | clou  (nail) | bague  (ring) |
| **fromage**  **(cheese)** | chèvre  (goat) | odeur  (smell) | beurre  (butter) | crème  (cream) | roi  (king) | peluche  (teddy bear) |
| **fusée**  **(rocket)** | espace  (space) | lune  (moon) | bateau  (ship) | métro  (metro) | chapeau  (hat) | lapin  (rabbit) |
| **gant**  **(glove)** | main  (hand) | hiver  (winter) | veste  (jacket) | chemise  (shirt) | coq  (rooster) | apéritif  (appetizer) |
| **girafe**  **(giraffe)** | cou  (neck) | savane  (savanna) | éléphant (elephant) | cochon  (pig) | bille  (ball) | radio  (radio) |
| **gomme**  **(eraser)** | effaçage  (erasing) | erreur  (mistake) | enveloppe (envelop) | carnet  (notebook) | oeil  (eye) | endormissement  (falling sleep) |
| **hache**  **(axe)** | coupe  (cut) | bûcheron  (lumberjack) | cisaille  (shear) | machette  (machete) | chaussure  (shoe) | moineau  (sparrow) |
| **harpe**  **(harp** | cordes  (strings) | mélodie  (melody) | piano  (piano) | tambour  (drum) | bateau  (ship) | cou  (neck) |
| **hérisson**  **(hedgehog)** | pique  (spine) | route  (road) | souris  (mouse) | âne  (donkey) | chiffon  (wipe) | poche  (pouch) |
| **journal**  **(newspaper)** | nouvelles  (news) | informations (informations) | radio  (radio) | téléphone (telephone) | roue  (wheel) | cerise  (cherry) |
| **kangourou**  **(kangaroo)** | poche  (pouch) | saut  (jump) | mouton  (sheep) | vache  (cow) | poussière  (dust) | ferme  (farm) |
| **lion**  **(lion)** | roi  (king) | crinière  (mane) | tigre  (tiger) | chat  (cat) | papier  (paper) | nouvelles  (news) |
| **lit**  **(bed)** | endormissement  (falling sleep) | sommeil  (sleep) | fauteuil (armchair) | table  (table) | neige  (snow) | crayon  (pencil) |
| **maison**  **(house)** | famille  (family) | toit  (roof) | immeuble  (building) | appartement (flat) | oeuf  (egg) | palmier  (palm tree) |
| **marteau**  **(hammer)** | clou  (nail) | tape  (hit) | tournevis  (screwdriver) | pince  (pliers) | souris  (mouse) | vol  (flight) |
| **moulin**  **(windmill)** | vent  (wind) | farine  (flour) | ferme  (farm) | château  (castle) | effaçage  (erasing) | sac  (bag) |
| **oreille**  **(ear)** | écoute  (hearing) | son  (sound) | visage  (face) | langue  (tongue) | noix  (walnut) | désert  (desert) |
| **ours**  **(bear)** | peluche  (teddy bear) | miel  (honey) | loup  (wolf) | cheval  (horse) | enveloppe (envelope) | cisaille  (shear) |
| **panier**  **(basket)** | osier  (wicker) | fruits  (fruit) | sac  (bag) | caisse  (box) | soleil  (sun) | brosse  (brush) |
| **papillon**  **(butterfly)** | ailes  (wings) | couleur  (color) | abeille  (bee) | moustique  (mosquito) | fourche  (fork) | noisette  (hazelnut) |
| **peigne**  **(comb)** | cheveux  (hair) | coiffure  (haircut) | brosse  (brush) | savon  (soap) | mare  (pond) | citron  (lemon) |
| **pelle**  **(shovel)** | sable  (sand) | creux  (hollow) | fourche  (fork) | tenaille  (bolt cutters) | fusil  (rifle) | vacances  (vacation) |
| **perceuse**  **(drill)** | bruit  (noise) | trou  (hole) | ciseaux  (scissors) | rouleau  (roller) | sable  (sand) | carton  (carton) |
| **pied**  **(foot)** | chaussure  (shoe) | marche  (walk) | jambe  (leg) | bras  (arm) | scooter  (scooter) | lait  (milk) |
| **pingouin**  **(penguin)** | banquise  (sea ice) | glace  (ice) | moineau  (sparrow) | aigle  (eagle) | boulet  (cannonball) | seau  (bucket) |
| **poêle**  **(frying pan)** | friture  (frying) | cuisine  (kitchen) | casserole  (pot) | verre  (glass) | mer  (sea) | singe  (monkey) |
| **poisson**  **(fish)** | mer  (sea) | aquarium (fishtank) | requin  (shark) | baleine  (whale) | feuille  (sheet) | mesure  (measure) |
| **poubelle**  **(dustbin)** | ordures  (garbage) | déchets  (waste) | carton  (carton) | bac  (tray) | friture  (frying) | loup  (wolf) |
| **raisin**  **(grape)** | vin  (wine) | jus  (juice) | cerise  (cherry) | pomme  (apple) | maths  (math) | boîte  (box) |
| **règle**  **(ruler)** | mesure  (measure) | trait  (line) | crayon  (pencil) | lettre  (letter) | champ  (field) | hélicoptère  (helicopter) |
| **scie**  **(saw)** | bois  (wood) | métaux  (metals) | tronçonneuse  (chain saw) | mètre  (meter) | écoute  (hearing) | beurre  (butter) |
| **ski**  **(ski)** | neige  (snow) | montagne (mountain) | patin  (ice skate) | traîneau  (sleigh) | casserole  (pot) | fleur  (flower) |
| **stylo**  **(pen)** | bille  (ball) | encre  (ink) | feuille  (sheet) | livre  (book) | requin  (shark) | bruit  (noise) |
| **tracteur**  **(tractor)** | champ  (field) | paysan  (farmer) | camion  (truck) | voiture  (car) | jupe  (skirt) | banquise  (sea ice) |
| **vase**  **(vase)** | fleur  (flower) | eau  (water) | boîte  (box) | pot  (jar) | cirque  (circus) | salade  (salad) |
| **vélo**  **(bike)** | roue  (wheel) | pédale  (pedal) | scooter  (scooter) | moto (motorcycle) | fourmi  (ant) | coupe  (cut) |
| **violon**  **(violin)** | musique  (music) | archet  (bow) | guitare  (guitar) | flûte  (flute) | camion  (truck) | jardin  (garden) |
